# Acute Peritoneal Dialysis in Critical Preterm Infants: A Case Series and Review of the Literature

**DOI:** 10.3390/children12091113

**Published:** 2025-08-24

**Authors:** Francesca Riitano, Serena Ferretti, Simonetta Costa, Eloisa Tiberi, Antonio Gatto, Giovanni Vento

**Affiliations:** 1 Dipartimento di Scienze della Salute della Donna, del Bambino e di Sanità Pubblica, Fondazione Policlinico Universitario Agostino Gemelli IRCCS, 00168 Rome, Italy; francesca.riitano@guest.policlinicogemelli.it (F.R.); serena.ferretti1@guest.policlinicogemelli.it (S.F.); eloisa.tiberi@policlinicogemelli.it (E.T.); antonio.gatto@policlinicogemelli.it (A.G.); giovanni.vento@unicatt.it (G.V.); 2 Catholic University of Sacred Heart, 00168 Rome, Italy

**Keywords:** acute kidney injury, kidney replacement therapy, peritoneal dialysis, preterm infants, newborn infants

## Abstract

**Background**: Acute kidney injury (AKI) in critically ill neonates is usually of pre-renal origin and, often, pharmacological treatment is not sufficient for resolution, requiring kidney replacement therapy (KRT). Due to the small body size and the unavailability of adequate devices for these patients, peritoneal dialysis (PD) appears to be the most easily achievable procedure. However, guidelines for PD management are lacking in this population. **Objective:** We aimed to report a single-center experience with preterm infants who underwent PD, describing the technical issues and the outcomes, and to review the existing literature. **Methods**: This retrospective study included preterm infants undergoing PD because of AKI unresponsive to pharmacological treatment. Data were compared to those available in the current literature. **Results**: Neonatal outcomes of twelve preterm infants were reported. PD was started before the onset of anuria in two oliguric patients, while it was started within 60 h of anuria in four patients, and between 72 and 144 h of anuria in the remaining six patients. One oliguric patient and one who started PD after 60 h of anuria had a complete recovery of kidney function with normalization of diuresis and renal function parameters. The other infants did not achieve complete resolution of AKI. The mortality rate was 91.7%, and even one of the two infants who had recovered kidney function later died due to an infectious complication. **Conclusions**: Our experience with a limited sample size did not allow us to obtain definitive conclusions. Our data and the current literature suggested that the prognosis is still negative, with a high mortality rate. Further research is needed to develop guidelines to optimize the management of preterm infants with AKI.

## 1. Introduction

Acute kidney injury (AKI) affects approximately 8–24% of critically ill neonates admitted to neonatal intensive care units (NICUs) [1]. AKI in newborn infants is more frequently of pre-renal than intrinsic kidney origin, due to prematurity, perinatal asphyxia, sepsis, acute respiratory distress syndrome, and patent ductus arteriosus [2]. Often, pharmacological treatment is not sufficient for resolution, and it is necessary to resort to kidney replacement therapy (KRT). In consideration of the small body size and complexity of these patients, as well as the unavailability of adequate devices for newborn infants, peritoneal dialysis (PD) appears to be the most easily achievable procedure [3]. This technique is not free from critical issues and requires careful control of electrolytes, fluids, acid–base balance, nutrition, and the prevention of infectious complications [4].

KRT should be started before the onset of irreversible kidney damage and excessive fluid overload [5]. However, the timing of PD starting in AKI is currently not defined by guidelines in the pediatric and neonatal populations, much less for the preterm infant population.

Working in a third-level NICU of a reference center hospital for high-risk pregnancies, we often find ourselves faced with patients with rapid clinical deterioration requiring multidisciplinary management. The aim of this study was to report our experience with premature infants undergoing PD, while also considering neonatal outcomes in relation to the timing of PD initiation.

## 2. Materials and Methods

### 2.1. Study Design, Setting, and Definitions

This retrospective study was performed in the NICU of Fondazione Policlinico Universitario Agostino Gemelli, IRCCS in Rome, Italy, between 1 January 2021 and 31 March 2025.

We analyzed clinical data from infants with gestational age (GA) < 37 weeks who developed AKI unresponsive to pharmacological treatment.

AKI was defined according to the Kidney Disease: Improving Global Outcomes (KDIGO) guidelines [6]. KDIGO neonatal criteria included serum creatinine elevation and/or urine output < 0.5 mL/kg/h for more than hours. Anuria was considered as urine output < 0.5 mL/kg/h for 24 h. Fluid overload (FO) was estimated using the fluid balance method and it was defined as %FO ≥ 10% over the baseline body weight (BW).

We extracted data from medical records. Details about GA, BW, clinical condition leading to the AKI, renal function parameters, timing of oligo-anuria, timing of initiation and duration of PD, and neonatal survival were collected.

### 2.2. Search Strategy for the Review of the Literature

In order to review the literature about the use of PD in preterm infants following AKI, an extensive literature search in the MEDLINE database (via PubMed) was performed up until 31 March 2025. The following keywords were also searched as entry terms: “peritoneal dialysis” OR “kidney replacement therapy” AND “preterm neonate” OR “preterm newborn” OR “preterm infant” AND “acute kidney injury” OR “renal failure”. All retrieved articles were screened by reading the abstract; if the abstract reported the use of PD, we assessed the full text for inclusion. References in the relevant papers were also reviewed, and further articles were added if necessary. Papers written in languages other than English were excluded. Available information was systematically collected.

## 3. Results

### 3.1. Case Series Presentation

During the study period we treated twelve preterm infants with AKI unresponsive to pharmacological treatment who underwent PD. Our cohort of patients was heterogeneous both in terms of basic characteristics (GA and BW) and in terms of the underlying conditions leading to the AKI (Table 1). There were eight extremely low birth weight infants, two low birth weight neonates, and two babies with a BW above 2500 g. The median (interquartile range) BW was 1842.5 g (787.5–1507.5) g. The median (interquartile range) GA was 28/2 (26/1–30/3) weeks/days. The underlying conditions were two twin-to-twin transfusion syndromes, one asphyxia, one non-surgical necrotizing enterocolitis, one sustained paroxysmal supraventricular tachycardia, four septic shock, and three fetal hydrops due to congenital anomalies.

In all infants, AKI presented with unstable hemodynamic conditions, weight gain, and dyselectrolytemia due to anuria. Only two infants were oliguric with FO. All children underwent pharmacological therapy with vasopressors, diuretics (furosemide and etacrynic acid), albumin, and fenoldopam at maximal dosage before starting PD.

The times for starting KRT were different in each individual case, and three infants required PD more than once (Table 2).

PD was started before the onset of anuria in the two oliguric patients, at 36 h of anuria in two patients, at 60 h of anuria in two patients, and between 72 and 144 h of anuria (average 94 ± 35 h) in the remaining patients. One oliguric patient and the one who started PD after 60 h of anuria had a complete recovery of kidney function with normalization of diuresis and renal function parameters. The other infants did not achieve complete resolution of AKI, and when a second PD course was started, it did not lead to improvements. The mortality rate was high at 91.7% and even one of the two infants who had recovered kidney function died due to an infectious complication (Table 2).

The dialytic procedures are detailed in Table 3. PD was performed in three cases with a surgically inserted Tenckhoff catheter, while in the other ones, according to the children’s low weight, we used percutaneously implanted pigtail mono-j 6F or 8F catheters. The PD fluids used were BicaVera (Fresenius Medical Care, Italy) in seven patients, and Balance (Fresenius Medical Care, Italy) in the other five; all PD fluids were medicated with antibiotic and antifungal drugs.

Dwell volume ranged between 10 and 50 mL, based on the patient capacity to tolerate peritoneal fluid load; dwell time ranged between 15 and 20 min.

### 3.2. Results of the Review of the Literature

Reviewing the existing literature, we found 20 papers reporting on 92 preterm infants who underwent PD after AKI, and the noteworthy data are summarized in Table 4 [1,3,7,8,9,10,11,12,13,14,15,16,17,18,19,20,21,22] and Table 5 [23,24].

The reported infants had median (interquartile range) GA of 26 (22–36) weeks, and median (interquartile range) BW of 710 (264–2405) g. The underlying conditions leading to AKI were different, while the device used as a catheter for PD was very different among the reported case series. The duration of PD ranged from 1 h to 117 days. No complete information regarding the time between the onset of oligo-anuria and the onset of PD was reported.

Regarding the outcomes of the 92 reported infants, 53 (57.6%) died, 33 (35.9%) survived or recovered, and 6 (6.5%) abandoned treatment.

## 4. Discussion

The small number of our case series did not allow us to draw relevant conclusions regarding the association between the time of onset of PD or other clinical variables and outcomes. The two infants who achieved complete resolution of kidney function were the more mature babies, and this may have been a positive prognostic factor for the recovery of kidney function. To settle how the GA may impact AKI resolution, a larger sample of patients is needed. Moreover, reviewing the existing literature reporting on preterm infants who underwent PD, we found no complete information regarding the time between the onset of oligo-anuria and the onset of PD; therefore, we cannot compare our renal and clinical outcomes in relation to the timing of PD initiation.

A critical issue is that there are no specific peritoneal catheters for newborn infants, because they are too large [3,24]. In premature infants, considering the low weight and the thick layer of subcutaneous tissue, a peritoneal drain could be positioned. In these tiny babies, the use of a straight dialysis catheter may heighten the risk of bowel perforation [24]. In contrast, the pigtail dialysis catheter may have a lower risk of bowel erosion. For this reason, the pigtail dialysis catheter is increasingly being adopted for use in neonates [24]. In our experience, we used the percutaneously implanted pigtail mono-j catheters in 75% of the infants, without catheter-related complications.

Another problem that may be encountered when managing this fragile population of infants is the hemodynamic and clinical instability. Clinical criticality often requires postponement of the placement of the peritoneal catheter for the start of dialysis, and this could negatively impact the clinical outcome.

The high complication rate, also described by our group during DP, represents a further critical point of the procedure for these patients with a negative impact on survival [3]. Death in our newborns occurred due to infectious complications in 50% or due to the evolution of the underlying clinical condition into multi-organ dysfunction in the remaining 50%.

Noh et al., who collected the largest case series in the literature of extremely low birth weight infants undergoing PD, started dialysis treatment after at least 48 h of anuria or in case of FO, refractory hyperkalemia, severe metabolic acidosis, and uremia, regardless of the hours of anuria, obtaining a high mortality (11 out of 12 patients) [17].

The mortality rate among our patients was 91.7%, which was very high and partially comparable to that reported in two of the largest case series by Stojanović et al. [1] and Noh et al. [17], at 75.0% and 91.6%, respectively. However, putting together all preterm infants who underwent PD reported in the literature to date, the mortality rate, which has been very high in the individual small case series, drops to 46.7%, with a recovery rate of renal function of 34.8%, indicating that the implementation of this procedure among preterm infants is desirable. The observed decline in mortality rate when all reported case series are pooled could be explained by the improved survival rate in case series with more mature infants.

## 5. Strengths and Limitations

This study had some strengths as well as some limitations that should be considered.

The main limitation of this study was the retrospective design and the small number of preterm infants included. The small sample of patients together with the high mortality prevented us from comparing the characteristics of the surviving newborn infants with those who did not.

However, considering that only 92 neonates undergoing PD have been reported to date, we believe that the experience on 12 neonates from a single-center NICU may provide insights into the management of preterm infants with AKI, representing, at the same time, the strength of this study. We strongly believe that clinical observations from case series, such as ours, and from observational clinical studies should be taken into account as starting point for the design of randomized clinical trials to generate evidence that can guide clinical decisions in preterm infants who develop AKI.

## 6. Conclusions

Our experience with a limited sample size did not allow us to obtain definitive conclusions. Our data and the review of the literature on premature infants who develop AKI highlighted that the prognosis is still negative, with a high mortality rate. The data collections on neonatal PD, even on a limited number of patients, are fundamental for understanding which types of patients can really benefit from this procedure and how to optimize the treatment. More research efforts on this emerging topic, such as multicenter clinical trials, are needed to improve clinical outcomes, given the high rate of complications and mortality. Further data may especially help to establish a precise and effective temporal cut-off for initiating PD.

## Figures and Tables

**Table 1:** Demographic and clinical characteristics of the twelve infants before the commencement of peritoneal dialysis.

Patient	GA Weeks/Days	BW Grams	Underlying Condition	BUN * mg/dL	Cr * mg/dL	K * mmol/L	Albumin * g/L
1	34/6	2800	PSVT	33	2.3	6.5	21
2	32/4	2600	Fetal hydrops	33	3.3	5.3	8
3	32/2	2250	Fetal hydrops	62	2.6	6.7	23
4	26/3	840	Non-surgical NEC	40	1.7	5.7	12
5	23/6	520	Septic shock	15	0.9	6.2	27
6	28/0	800	TTTS	90	4.2	6.3	19
7	29/1	750	TTTS	15	3.2	2.5	23
8	25/2	880	Perinatal asphyxia	33	1.8	7.9	17
9	28/2	1260	Fetal hydrops	50	1.4	6.4	30
10	23/1	660	Septic shock	25	1.55	6.9	17
11	27/1	1000	Septic shock	85	1.78	5.1	35
12	26/3	885	Septic shock	63	0.63	6.6	17

GA: gestational age; BW: birth weight; BUN: blood urea nitrogen; Cr: creatinine; K: potassium; NEC: NEC: necrotizing enterocolitis; PSVT: paroxysmal supraventricular tachycardia; TTTS: twin-to-twin transfusion syndrome. * BUN, Cr, K, and albumin values refer to the time before the commencement of peritoneal dialysis.

**Table 2:** Peritoneal dialysis course and outcomes of the twelve infants with acute kidney injury.

Patient	PD Onset # Hours	PD Duration Days	PD Restarts Number	BUN at Resolution mg/dL	Cr at Resolution mg/dL	K at Resolution mmol/L	Recovery of KF	Death	Cause of Death
1	/	31	1	37	0.6	4.2	Yes	No	/
2	60	13	/	30	0.9	4.4	Yes	Yes	Septic shock
3	72	87	3	/	/	/	No	Yes	Septic shock
4	72	13	/	/	/	/	No	Yes	MODS
5	72	3	/	/	/	/	No	Yes	Septic shock
6	144	103	2	/	/	/	No	Yes	Septic shock
7	144	18	/	/	/	/	No	Yes	Heart failure
8	60	3	/	/	/	/	No	Yes	MODS
9	36	3	/	/	/	/	No	Yes	Heart failure
10	60	21	/	/	/	/	No	Yes	Septic shock
11	/	16	/	/	/	/	No	Yes	Septic shock
12	36	18	/	/	/	/	No	Yes	MODS

BUN: blood urea nitrogen; Cr: creatinine; K: potassium; KF: kidney function; MODS: multiple organ dysfunction syndrome; PD peritoneal dialysis.

**Table 3:** Dialysis treatment details.

Patient	Catheter Type	PD Solution Type	Antibiotics in PD Solution	Dwell Volume (mL)	Dwell Time (min)
1	Pigtail mono-J	Balance 1.5%	Ceftazidime Fluconazole	50	15
2	Tenckhoff	BicaVera 1.5%	Ceftazidime Fluconazole	20	15
3	Pigtail mono-J	Balance 1.5%	Ceftazidime Fluconazole	20	15
4	Pigtail mono-J	Balance 1.5%	Ceftazidime Fluconazole	20	15
5	Pigtail mono-J	BicaVera 1.5%	Ceftazidime Fluconazole	20	20
6	Pigtail mono-J	BicaVera 2.3%	Ceftazidime Fluconazole	10	15
7	Pigtail mono-J	Balance 1.5%	Ceftazidime Fluconazole	20	15
8	Tenckhoff	BicaVera 2.3%	Ceftazidime Fluconazole	20	20
9	Pigtail mono-J	BicaVera 2.3%	Ceftazidime Fluconazole	20	15
10	Pigtail mono-J	BicaVera 2.3%	Ceftazidime Fluconazole	20	15
11	Pigtail mono-J	Balance 1.5%	Ceftazidime Fluconazole	40	20
12	Pigtail mono-J	BicaVera 2.3%	Ceftazidime Fluconazole	30	20

**Table 4:** Case series describing preterm infants who underwent peritoneal dialysis.

Authors	GA Weeks/Days	BW Grams	Cause of AKI	Catheter Type	Insertion Site	Age at the Beginning of PD (Days)	Duration of PD	Complications	Outcome
Stojanović et al. [1]	25/0	690	Sepsis	IV cannula	Left side of umbilicus	6	3 h	None	Dead
Stojanović et al. [1]	27/1	470	Sepsis	IV cannula	Left side of umbilicus	4	2.5 h	Leakage	Dead
Stojanović et al. [1]	27/3	890	Gentamicin	IV cannula	Left side of umbilicus	17	28 h	None	Dead
Stojanović et al. [1]	27/0	880	Sepsis	UVC	Left side of umbilicus	28	2 days	Peritonitis	Dead
Stojanović et al. [1]	27/0	610	Sepsis	IV cannula	Left side of umbilicus	11	4 h	Obstruction	Dead
Stojanović et al. [1]	25/0	880	NEC, PDA	IV cannula	Left side of umbilicus	2	21 h	None	Recovered
Stojanović et al. [1]	25/0	870	Apnea	IV cannula	Left side of umbilicus	13	1.5 h	Leakage	Dead
Stojanović et al. [1]	25/0	700	Asphyxia	IV cannula	Left side of umbilicus	20	22 h	None	Recovered
Gatto et al. [3]	28/0	800	TTTS	10 Fr single-cuff Foley catheter, then mono-j straight 6F catheter	Left iliac fossa	6	117 days	Peritonitis, leakage	Dead
Alparslan et al. [7]	28/0	1000	RDS	Tenckhoff, one-cuffed neonatal catheters with straight tips	Through the linea alba toward the left iliac fossa	ND	12 days	None	Dead
Ao et al. [8]	24/3	700	Sepsis	14-gauge CVC	1 cm lateral left of the umbilicus	1	14 h	ND	Recovered
Ao et al. [8]	24/6	750	ARDS	14-gauge CVC	1 cm lateral left of the umbilicus	3	1.5 days	ND	Recovered
Ao et al. [8]	27/1	880	Asphyxia	14-gauge CVC	1 cm lateral left of the umbilicus	1	2 days	ND	Recovered
Burgmaier et al. [9]	22/0	430	Sepsis	Ascites drainage catheter	ND	120	19 days	Hyperglycemia	Dead
Burgmaier et al. [9]	23/0	614	IFI	Drainage catheter, then PD catheter	ND	7–14	44 days	Leakage, catheter dislocation, peritonitis, catheter obstruction, hyperglycemia	Recovered
Çetinkaya et al. [10]	24/0	460	Sepsis	PD catheter	Left paramedian above umbilicus	12	6 days	Leakage	Dead
Chen et al. [11]	23/5	650	Sepsis	Arrow catheter	McBurney point	10	3 days	Leakage	Recovered
Harshman et al. [12]	28 + 3	830	TTTS	PD catheter	Left upper quadrant	5	16 days	None	Recovered
Jiang et al. [13]	31/2	1720	Sepsis	PD catheter	Left side of the abdomen	10	19 days	Bowel prolapse	Recovered
Kanarek et al. [14]	25/0	710	Asphyxia	Trocath-McGaw catalog #V4900	ND	2	30 h	None	Recovered
Kaul et al. [15]	27/0	960	NEC	Intercostal drain (modified)	Left paraumbilical region	40	2 days	None	Recovered
Macchini et al. [16]	28/0	630	Sepsis	single-cuff Tenckhoff catheter	Paramedian entry-site	10	27 days	Leakage	Recovered
Noh et al. [17]	25/1	960	Sepsis	PD catheter	McBurney point	8	9.4 (5–14) days	Leakage, intraperitoneal hemorrhage, obstruction	Dead
Noh et al. [17]	26/4	990	MODS	UVC	McBurney point	3	9.4 (5–14) days	Peritonitis, intraperitoneal hemorrhage	Dead
Noh et al. [17]	25/2	420	Sepsis	Arrow catheter	McBurney point	43	9.4 (5–14) days	None	Dead
Noh et al. [17]	24/2	540	Sepsis	Arrow catheter	McBurney point	26	9.4 (5–14) days	Leakage, obstruction	Dead
Noh et al. [17]	26/6	760	Sepsis	PD catheter	McBurney point	43	9.4 (5–14) days	Leakage	Dead
Noh et al. [17]	26/6	550	PH	Arrow catheter	McBurney point	3	9.4 (5–14) days	Leakage	Dead
Noh et al. [17]	23/6	470	Bilateral renal vein thrombosis	PD catheter	McBurney point	129	9.4 (5–14) days	Leakage	Dead
Noh et al. [17]	26/3	868	Bilateral renal vein thrombosis	PD catheter	McBurney point	134	9.4 (5–14) days	Leakage, intraperitoneal hemorrhage	Dead
Noh et al. [17]	27/6	590	Cardiogenic shock, TTTS	PD catheter	McBurney point	3	9.4 (5–14) days	None	Dead
Noh et al. [17]	27/3	470	Sepsis	PD catheter	McBurney point	13	9.4 (5–14) days	Obstruction	Recovered
Sizun et al. [18]	28/0	680	Sepsis	Venflon Viggo 16-gauge peripheral venous catheter	ND	41	2 days	None	Dead
Sizun et al. [18]	25/0	700	Asphyxia	Venflon Viggo 16-gauge peripheral venous catheter	ND	5	3 days	None	Dead
Ustyol et al. [19]	26/0	600	Sepsis	PD catheter	0.5–1 cm below the umbilicus	14	3 days	ND	Dead
Ustyol et al. [19]	25/0	750	Sepsis	PD catheter	0.5–1 cm below the umbilicus	11	1 days	ND	Dead
Ustyol et al. [19]	24/0	580	Sepsis	PD catheter	0.5–1 cm below the umbilicus	7	3 days	ND	Dead
Ustyol et al. [19]	24/0	700	Sepsis	PD catheter	0.5–1 cm below the umbilicus	8	1 days	ND	Dead
Yildiz et al. [20]	32/0	1820	CDH	PD catheter	Left lower quadrant	10	10 days	Non	Dead
Yokoyama et al. [21]	24/0	264	Sepsis	Drainage catheter	Right umbilical region	21	32 days	Leakage, peritonitis	Recovered
Yu et al. [22]	26/0	930	Sepsis	14-gauge arrow vascular catheter	Iliac fossa	10	2 days	Hernia	Recovered
Yu et al. [22]	26/0	890	PDA	14-gauge arrow vascular catheter	Iliac fossa	25	2 days	Peritonitis	Recovered
Yu et al. [22]	28/0	900	Sepsis	14-gauge arrow vascular catheter	Iliac fossa	14	6 days	None	Recovered
Yu et al. [22]	28/0	730	Sepsis	14-gauge arrow vascular catheter	Iliac fossa	26	3 days	None	Recovered
Yu et al. [22]	26/0	680	PDA	14-gauge arrow vascular catheter	Iliac fossa	28	6 days	None	Recovered
Yu et al. [22]	27/0	690	PH	14-gauge arrow vascular catheter	Iliac fossa	8	8 days	None	Recovered
Yu et al. [22]	26/0	700	IVH	14-gauge arrow vascular catheter	Iliac fossa	21	2 days	Leakage	Dead
Yu et al. [22]	28/0	980	Sepsis	14-gauge arrow vascular catheter	Iliac fossa	32	3 days	Peritonitis	Dead
Yu et al. [22]	26/0	840	NEC	14-gauge arrow vascular catheter	Iliac fossa	18	2 days	Leakage, hemoperitoneum	Dead
Yu et al. [22]	26/0	630	PH	14-gauge arrow vascular catheter	Iliac fossa	27	2 days	None	Dead
Yu et al. [22]	24/0	820	PDA	14-gauge arrow vascular catheter	Iliac fossa	26	3 days	None	Dead

BW: birth weight; GA: gestational age; PD: peritoneal dialysis; AKI: acute kidney injury; IV: intravenous; UVC: umbilical venous catheter; CVC: central venous catheter; TTTS: twin-to-twin transfusion syndrome; RDS: respiratory distress syndrome; ARDS: acute respiratory distress syndrome; CDH: congenital diaphragmatic hernia; IFI: invasive fungal infection; PDA: patent ductus arteriosus; NEC: necrotizing enterocolitis; PH: pulmonary hemorrhage; IVH: intraventricular hemorrhage; MODS: Multiple organ dysfunction syndrome; ND: not defined.

**Table 5:** Observational study describing preterm infants who underwent peritoneal dialysis.

Authors	Number of Infants	GA Weeks/Days	BW (Grams)	Cause of AKI	Catheter Type	Insertion Site	Age at the Beginning of PD (Days)	Duration of PD	Complications	Kidney Recovery Rate (%)	Mortality Rate (%)
Tangirala et al. [23]	20	32.6 ± 4.0	1500 (983–2405)	Sepsis, asphyxia, MODS, hypoxemia	Pigtail catheter Tenckhoff and Romson’s catheter	Infra-umbilical or a point midway between the umbilicus and anterior superior iliac spine on either side	6 (2–76)	3.4 ± 1.6 days	Catheter obstruction Catheter leakage Peritonitis	27	60
Xing et al. [24]	21	28.9 ± 2.6	1226.7 ± 495.3	Sepsis asphyxia, TTTS, arrythmia, edema	14 F gastric tube, 10F suction tube, neonatal PD catheter.	At the umbilicus, at 1 cm to the left of the umbilicus, at 1 cm to the right of the umbilicus, to the left of McBurney’s point.	4	3 days (1 h-20 days)	Inadequate drainage Drainage infection	33.3	38.1

BW: Birth weight; GA: gestational age; PD: peritoneal dialysis; AKI: acute kidney injury; TTTS: twin-to-twin transfusion syndrome; MODS: multiple organ dysfunction syndrome.

## Data Availability

All data considered for this case report have been included in this article. Articles considered for the review of the literature are already available on PubMed.
